# Sensitive Monitoring of the Minimum Inhibitor Concentration under Real Inorganic Scaling Scenarios

**DOI:** 10.1021/acsomega.4c04912

**Published:** 2024-08-15

**Authors:** Vitória M. S. Freitas, Waldemir J. Paschoalino, Luis C. S. Vieira, Jussara M. Silva, Bruno C. Couto, Angelo L. Gobbi, Renato S. Lima

**Affiliations:** † Brazilian Nanotechnology National Laboratory, Brazilian Center for Research in Energy and Materials, Campinas, São Paulo 13083-970, Brazil; ‡ Leopoldo Américo Miguez de Mello Research and Development Center, Petrobras, Rio de Janeiro, RJ 21941-598, Brazil; § Institute of Chemistry, University of Campinas, Campinas, São Paulo 13083-970, Brazil; ∥ Federal University of ABC, Santo André, São Paulo 09210-580, Brazil; ⊥ São Carlos Institute of Chemistry, University of São Paulo, São Carlos, São Paulo 09210-580, Brazil

## Abstract

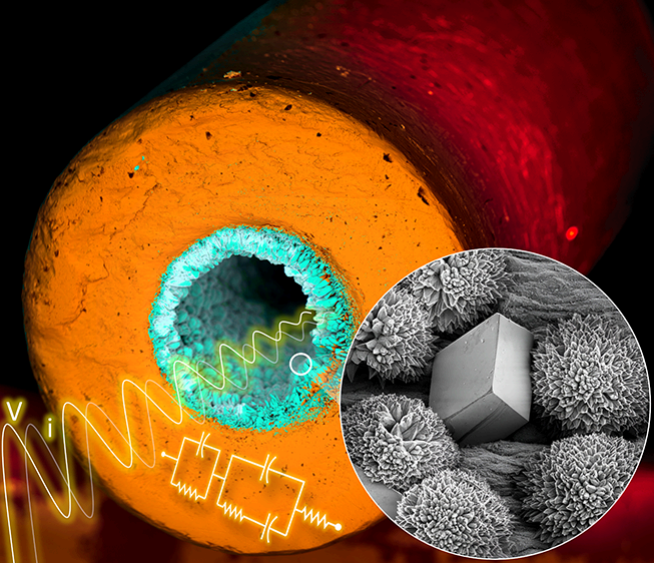

Flow assurance is a long-term challenge for oil and gas exploration as it plays a key role in designing safe and efficient operation techniques to ensure the uninterrupted transport of reservoir fluids. In this regard, the sensitive monitoring of the scale formation process is important by providing an accurate assessment of the minimum inhibitor concentration (MIC) of antiscale products. The optimum dosage of antiscale inputs is of pivotal relevance as their application at concentrations both lower and higher than MIC can imply pipeline blockages, critically hindering the entire supply chain of oil-related inputs and products to society. Using a simple and low-cost impedimetric platform, we here address the monitoring of the scale formation on stainless-steel capillaries from its early stages under real topside (ambient pressure and 60 °C) and subsea (1000 psi and 80 °C) sceneries of the oil industry. The method could continuously gauge the scale formation with a sensitivity higher than the conventional approach, i.e., the tube blocking test (TBT), which proved to be mandatory for avoiding misleading inferences on the MIC. In fact, whereas our sensor could entail accurate MICs, as confirmed by scanning electron microscopy, TBT suffered from negative deviations, with the predicted MICs being lower than the real values. Importantly, the impedance measurements were performed through a hand-held, user-friendly workstation. In this way, our method is envisioned to deliver an attractive and readily deployable platform to combat the scale formation issues because it can continuously monitor the salt precipitation from its early stages and yield the accurate determination of MIC.

## 1 Introduction

While global climate claims for new and clean sources of energy, the world is still a few decades away from completely turning or shutting down fossil fuels of our life. Hence, apart from the crucial efforts that have been made toward less-carbon emissions, research aiming at more efficient processes in the oil and gas industry is of pivotal importance since this well-established energy source will continue as the primary global energy matrix for the coming years.^1^ Specifically, oil is expected to provide 28% of energy needs worldwide by 2045.^2−4^ One should also emphasize that oil is extensively used in the production of diverse industrial goods of daily use, such as plastics and lubricants.

Over the past decades, the oil and gas industry has spent billions of dollars on flow assurance (FA) research. FA is especially challenging in deep-water offshore systems as the conditions of scale formation are more favorable.^5,6^ Scale formation is one of the main problems in ensuring ideal flow regimes along the oil extraction, processing, and transportation, critically impacting the efficiency and cost of operations, and being detrimental to the entire supply chain of related inputs and products to society. This phenomenon means the precipitation of various inorganic salts and it can happen in the reservoirs, downhole equipment, and wellhead during oilfield operations.^7−9^ The precipitation of calcium carbonate (CaCO_3_) is one of the most common scale formation phenomena in the reservoirs and producing wells, being caused by different reasons.^10−13^ One of them is if the concentration of ions exceeds their equilibrium content.

During oil extraction, the mixing between the injection and formation waters leads to the production of insoluble salts, disturbing the dynamic equilibrium in the reservoir. This equilibrium offset is sensitively affected by changes in the pH, partial pressure, water ionic composition, and temperature, providing the ideal conditions for scale formation.^14^ This salt accumulation shrinks the inner diameter of the production path, leading to a loss in the oil carrying capacity, pipe surface overheating, and an increase in operational costs.^15^ In this regard, the use of approaches capable of monitoring the scale formation process can be a game changer for the petrochemical industry in its long-standing striving to secure ideal flow conditions. Specifically, this type of information allows a comprehensive study on the efficiency of scale inhibitors, thus assisting (i) the adoption of optimum dosages of off-the-shelf products for specific operation conditions, places, and periods of time and (ii) the creation/development of new antiscale inputs. Ideally, the sensing method should deliver sensitive monitoring into daily practice in a continuous, in situ, and real-time way. The kinetic information provided by such methods can be useful to uncover scale formation mechanisms, further aiding the creation of antiscale products.

The most used approach to monitor scale formation and determine the minimum inhibitor concentration (MIC) is the tubing blocking test (TBT), which simulates the conditions in oil processing operations to probe pressure changes along scale formation.^16−18^ This method is based on the flow-assisted mixing of synthetic injection and formation waters, with resulting heterogeneous precipitation of insoluble salts on specific tubes of interest, such as stainless-steel (SS) capillaries. As a limitation, the early stages of scale formation (i.e., the nucleation processes followed by the deposition of the first on-capillary salt layers) cannot be detected through TBT as changes in the total pressure of the system are only recorded after an extensive salt deposition (it is demonstrated herein). This poor sensitivity not only precludes kinetic investigations but can also critically imply misleading inferences on the optimum MIC to combat scale formation.

Electrochemical impedance spectroscopy (EIS) has increasingly proven to be a powerful tool for diverse applications in various research and technology sectors.^19^ Recently, some articles demonstrated the use of this technique to assess the scale formation in refrigeration^20,21^ and reverse osmosis systems,^22,23^ along with the characterization of mineral deposition,^24^ presenting very attractive results by providing sensitive, *in*-*situ*, and real-time monitoring. Here we address a mesofluidic platform integrating an EIS-based sensor to provide low-cost, sensitive, and continuous assessment of the scale formation kinetics on SS capillaries, along with the determination of the real MIC in oil exploration industry sceneries. The SS capillaries acted as polarizable electrodes to be interrogated with impedance (Z) measurements. EIS produces chemically diversified Z vs frequency spectra, with distinct phenomena being detected that include (i) the interface accumulation of charges on clean and passivated electrodic surfaces (capacitive) and (ii) the transport of ions through liquid and solid phases (resistive phenomenon).^25−28^ These two types of impedimetric responses acted cooperatively to allow us to correlate the real-time changes in Z with gradual scale formation on SS since its early stages.

As a first step, we present a detailed study of the impedimetric responses from sensors before and along scale formation using a mild brine scenery for electric field-mediated deposition of CaCO_3_. Using a three-electrode setup composed of conventional reference (RE) and counter electrodes (CE) with the SS capillaries operating as working electrodes (WE), this investigation was intended (i) to assess the relationship between the inorganic scale formation and Z, and (ii) to define the working frequency for the next tests.

Second, we demonstrated the method’s applicability in a real topside scenery of deep-water–oil exploration by mixing specific formation (cationic) and injection (anionic) brines that implied the natural precipitation of CaCO_3_ salts. These analyses were conducted under ambient pressure in the absence and presence of two off-the-shelf antiscale products. A Y-shaped mesofluidic device was mounted with PEEK (polyetheretherketone) junctions for mixing the solutions. Focused on further streamlining the sensor, a two-electrode fashion was developed based on the adoption of SS capillaries as polarizable electrodes, WE and CE. We adjusted the Z data to an exponential fitting to assess the kinetic behavior of on-SS CaCO_3_ precipitation, providing us with information on the more effective antiscale input. It is also worthwhile emphasizing that the platform was further comprised of a pump and a voltage-assisted heating system.

To ensure a rigorous comparison of our sensor (two-electrode fashion) with conventional TBT, both Z and pressure measurements were next hyphenated in the scale formation experiments. In this case, we used cationic and anionic brines that led to the precipitation of Calcium (CaSO_4_), Barium (BaSO_4_), and Strontium (SrSO_4_) sulfate salts. Apart from the topside scenery, the method was successfully challenged in a real subsea scenery (1000 psi and 80 °C), therefore revealing the possibility of using the system at high-pressure conditions in the oil industry as well. Both approaches were able to monitor the time needed for complete salt-induced construction of the SS capillaries, while the impedimetric sensor provided more sensitive monitoring. With the aid of SEM images, the real-time changes in Z could be correlated with the salt precipitation over time, supporting how valuable our platform can be for the oil industry by providing the dosage of real MICs and, thus preventing the issues caused by stopping oil production due to pipeline blockage.

## 2 Materials and Methods

### 2.1 Three-Electrode Impedimetric Sensor and Platform

The mesofluidic electrochemical device was first assembled using polypropylene Luer fittings (Cole Parmer, US) to insert the electrodes, i.e., WE, RE, and CE, and define the brine flow outlet. SS 316 capillaries with an inner diameter (id) of 1 mm were used as WE, whereas a platinum wire and Ag/AgCl in 3.0 mol L^–1^ KCl (Metrohm, Switzerland) acted as CE and RE, respectively. Electrochemical analyses were performed on a portable Palmsens4 potentiostat. Closed-loop flow system was mounted with standard seawater (ASTM D1141), without potassium (KCl) and magnesium chloride (MgCl_2_), passing through a peristaltic pump, following to SS heat exchanger (HE), entering the device, and returning to the brine flask. The composition of this seawater is described in Table 1 and more information on this three-electrode system is available in the Supporting Information (Figure S1).

**Table 1:** Brine Composition^29^ along the Assays Using the Three-Electrode Sensor

**salt**	**concentration**(mol L^–1^)
NaCl	0.4
CaCl_2_	1.0 × 10^–2^
Na_2_SO_4_	2.8 × 10^–2^
NaHCO_3_	2.8 × 10^–3^

### 2.2 Scale Formation by Electrodeposition

CaCO_3_ scale was induced by chronoamperometry applying a constant potential of −1.1 V for 120 or 180 min. The scale formation was monitored by electrochemical impedance spectroscopy (EIS) measurements at predetermined times. A direct current potential (E_DC_) of −0.2 V and an alternating current potential (E_AC_) of 100.0 mV were applied in a frequency range of 1.0 to 5 × 10^4^ Hz. Z and constant phase angle (Φ) data were used for scale growth verification after 5, 10, 30, 60, 90, 120, 150, and 180 min of electrochemical deposition of CaCO_3_. Experiments were also made utilizing a scale inhibitor provided by Petrobras (designated as Product A), which was added directly to the standard seawater solution.

### 2.3 Two-Electrode Sensor in a Real Topside Scenery

The electrochemical sensor was challenged in a real scenario using the principles of TBT, e.g., with spontaneous scale formation after mixing of cationic and anionic brines under ambient pressure. These media were initially brought together in an SS Y-connection, then flowing through a 10 cm tube (CE), a PEEK junction, and a 5 cm tube (WE). Both electrodes were made of SS 316 capillaries with 1 mm id. The PEEK junction was used to promote electrical insulation between the two capillaries (gap of 5 mm). The experimental apparatus and more information on the setting with this two-electrode sensor (Figure S2) are available in the Supporting Information. The scale formation was promoted by simply mixing cationic and anionic brines in an open system where the outlet brine does not return to the initial bottle. The Z values were recorded over time by fixing the frequency at 5 × 10^4^ Hz. The composition of brines (Table S1) and experimental conditions (60 °C and pH 7.4) were applied as recommended by Petrobras. Measurements were made in the absence and presence of Product A and another off-the-shelf scale inhibitor (designated as Product B) that was supplied by Petrobras as well.

### 2.4 Hyphenation of Our Sensor with TBT in Real Topside and Subsea Scenarios

Pressure and impedance measurements were made in the same experiment using an HPLC pump (LC 40D, Shimadzu) to move cationic and anionic brines. The pressure values were collected from the pump and the EIS analyses were made over time as the previous routine (under ambient pressure) when employing the two-electrode sensor. As a single modification, the WE consisted of SS capillary wrapped into a spiral shape bearing 0.5 mm id and 100 cm length (Figure S3). In the hyphenated analyses, only Product B was first employed, and a composition of cationic and anionic brines provided by Petrobras (confidential information) was adopted. To further assess the reliability of the platform, monitoring at a harsh pressure condition (1000 psi) was also conducted (at 80 °C) by coupling a backpressure valve (Swagelok) at the end of the system. In this case, another antiscale product provided by Petrobras (Product C) was used in the subsea scenery.

### 2.5 Scale Characterization

Assays by field emission gun scanning electron microscopy (FEG-SEM) and energy dispersive X-ray spectrometry (EDS; Scientific Inspect F50, Thermo Fisher) were made to determine the shape and composition of the distinct morphologies of crystals, i.e., calcite, aragonite, and vaterite. Each one of these morphologies was identified by RAMAN spectroscopy (Spectrometer Xplora Plus Horiba). All the samples were prepared using a polishing machine until the SS capillaries showed roughly a hollow half-cylindrical shape.

## 3 Results and Discussion

### 3.1 Platforms

**Scheme 1:**
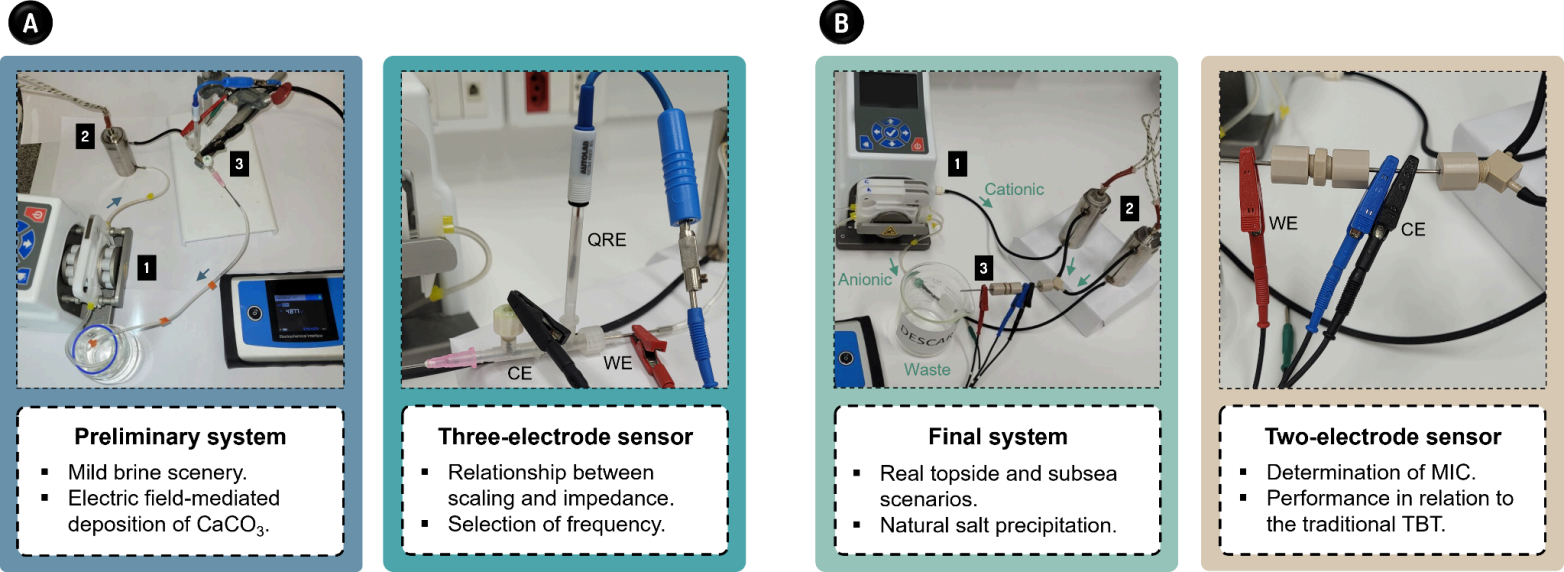
(A) Closed Flow System Bearing Three-electrode Sensor to Probe the Formation of CaCO_3_ Scale and (B) Two-Electrode System for Spontaneous Formation of CaCO_3_ scale In both images, (1), (2), and (3) identify the peristaltic pump, heat exchanger, and sensor, respectively.

CaCO_3_ scale formation was first achieved by electrodeposition, being monitored through a three-electrode sensor coupled to a closed flow system, as displayed in Scheme 1A. Brine passes through an SS 304 capillary tube (WE), and then RE and CE until it reaches the outlet, returning to the initial bottle. Second, to challenge the method’s applicability realistically, the impedimetric sensor was used in the monitoring of CaCO_3_ deposition triggered by simply mixing anionic and cationic brines, dispensing the prior electrochemical step to induce salt precipitation onto SS capillaries. The scale formation was obtained at 60 °C, pH 7.4, and ambient pressure, and the two brines were pumped through the HE and then mixed in a Y-shaped connection before flowing into polarizable SS electrodes to perform the impedimetric analyses, as exposed in Scheme 1B. For the hyphenation of our sensor with TBT in real scenarios, we replace the peristaltic pump and WE (see Scheme 1B) for a HPLC pump and a SS capillary with 0.5 mm id and 100 cm length, respectively (see Figure S3).

### 3.2 Three-Electrode Sensor to Monitor CaCO_3_ Scale

#### 3.2.1 Electrodeposition

A spontaneous scaling in SS capillaries would take too long due to the low salt concentration in the brine. Therefore, an electrochemical deposition was carried out to induce the scale in the three-electrode setup by chronoamperometry at negative potentials. This process is based on oxygen reduction (ORR) and hydrogen evolution (HER) reactions in a neutral environment, represented by eqs 1, 2 and 3, respectively.
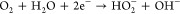
1
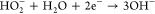
2
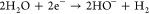
3

All these reactions lead to OH^–^ ion formation, raising the local pH at WE surroundings and ultimately inducing the CaCO_3_ precipitation as this process is favored at alkaline media.^29^ Such reactions are usually slow and depend on pH, catalyst, and adsorption processes.^30,31^

**Figure 1:**
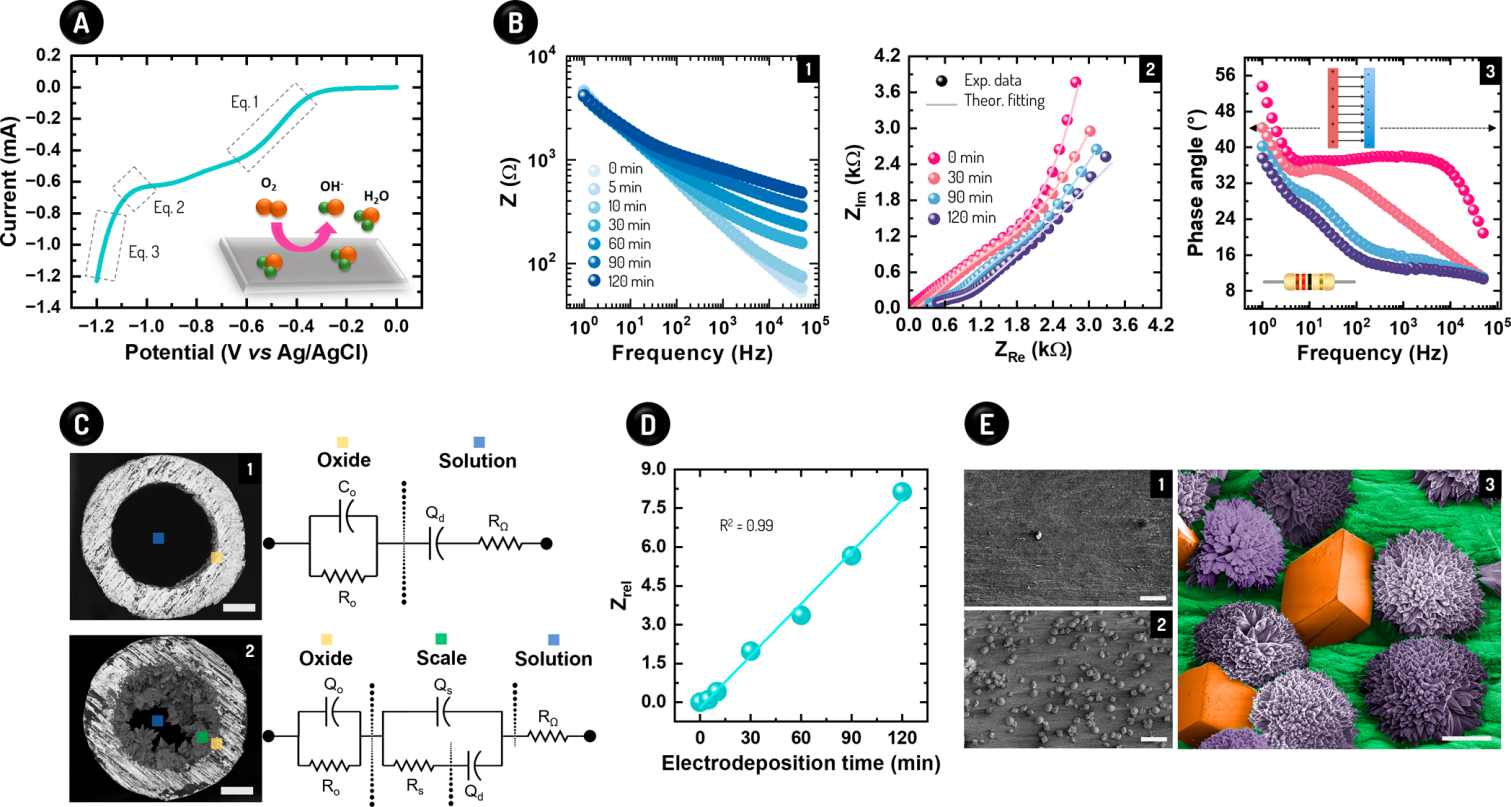
Electrodeposition method for scale formation and detection principle. (A) Linear sweep voltammogram of SS capillary and a schematic representation of the reactions on its surface. (B) Bode plot in a frequency range from 10° to 5 × 10^4^ Hz over time (1), Nyquist plot with experimental (Exp.) data and theoretical (Theor.) fitting (2), and phase angle plot with illustrations of capacitive and resistive phenomena above and below 45°, respectively (3). Z_Im_ and Z_Re_ mean real imaginary impedances, respectively. (C) Equivalent circuits for the SS surfaces before (1) and along the electrodeposition (2). The images showing the SS cross-section are merely illustrative, as they were reached by mixing cationic and anionic brines, without applying the electrodeposition procedure. The scale bars mean 200 μm (1,2). (D) Curve of Z_rel_ vs electrodeposition time. (E) SEM image of the CaCO_3_ crystals before (1) and after (2) 120 min of electrodeposition at a flow rate of 0.2 mL min^–1^, along with a high-magnitude image of the scale formation showing calcite (orange) and aragonite (purple) polymorphs (3). The scale bars correspond to 50 (1,2) and 10 μm (3).

The electrochemical characterization of the device was performed by linear sweep voltammetry, as shown in Figure 1A. The prior reactions are especially expected to occur at the potentials between −0.4 and −0.6 V (1) and between −1.1 and −1.2 V vs Ag/AgCl (2 and 3).^29^ While the reactions 1 and 3 imply, respectively, the production of low amount of OH^–^ ions and H_2_ evolution, the reaction 2 leads to the largest OH^–^ concentration. Thereby, the potential of −1.1 V (related to reaction 2) was chosen to perform the following electrodeposition experiments, which proceeded for 120 or 180 min.

#### 3.2.2 Impedimetric Sensor and Frequency Optimization

The scale formation was monitored by EIS at several predetermined times during the electrodeposition, i.e., 5-, 10-, 30-, 60-, 90-, and 120 min. Different time measurements were applied to evaluate the deposited salt over time, providing insights into the kinetic behavior of the scale formation using an impedimetric sensor. Figure 1B presents the Bode, Nyquist, and Φ plots for a flow rate of 1.0 mL min^–1^; such flow rate was chosen to understand the impedance behavior over the on-capillary salt deposition. The Z data increased over time, especially at high frequencies, signaling the gradual precipitation of the CaCO_3_ scale on SS capillary surfaces. EIS provides information about the resistive and capacitive phenomena that drive the Z data. In practice, this task is achieved by fitting RC equivalent circuits to the experimental Z values,^28^ as shown in Figure 1C. The analysis of their components allowed us to understand the changes from bulk to the electrical double layer (EDL) of WE along the electrodeposition, as discussed next.

Two circuits fitted well the experimental Z values for the WE before and after the electrodeposition of CaCO_3_ (see Figure 1B,C and Table S2). The capacitances were represented as a function of constant phase elements (CPE), indicating that the capacitors did not behave as true electrical elements due to the high roughness of SS capillary surfaces. Before the scale formation, the circuit showed the solution resistance (R_Ω_) in series with the EDL capacitance (Q_d_), which are in parallel with elements (R_o_ and Q_o_) related to the oxide layer that is found on the inner walls of off-the-shelf SS capillaries.^32^ Once the electrodeposition started, a contribution from scale formation was added between the two prior parts (oxide and EDL/solution), being represented by RC elements (R_s_ and Q_s_) in parallel (see Figure 1C).

Most Φ data ranged from 10° up to 40° (see Figure 1B), showing that the Z values depended on both capacitive (charge accumulation at solid/solid and solid/liquid interfaces) and resistive (movement of mobile charges through solid and liquid phases) responses. Nonetheless, one should underline that the contribution of the resistive phenomena for the impedances was significantly higher than the capacitive contribution (Φ < 45°), especially at the high frequencies.^26,28^ In this regard, the increase in Z with the progress of CaCO_3_ electrodeposition can be ascribed to the gradual enhancement in R_Ω_, as R_o_ and R_s_ reduced over time (Figure S4). The solution became more resistive due to the continuous depletion of Ca^2+^ and CO_3_^–^ ions, as confirmed by analyses using a Ca^2+^ selective electrode (Table S3). These measurements revealed a decrease of 10.6 ± 1.6% in the Ca^2+^ concentration in brine about its initial concentration (10.0 mmol L^–1^) after 180 min of electrodeposition. Therefore, the depletion of Ca^2+^ and CO_3_^–^ ions in our closed flow platform along the scale formation is supposed to increase R_Ω_, ultimately leading to increased values of Z.

While the Z data were also governed by high values of R_o_ and R_s_ at the early stages of scaling, these parameters reduced over time as mentioned before (see Figure S4). The decrease in R_o_ can be explained by the reducing potential (i.e., – 1.1 V) that was applied for the CaCO_3_ electrodeposition. Such a voltage is expected to partially remove the oxide while incrustation occurs. This interface phenomenon is likely the cause of the decrease in charge accumulation through the oxide layer from 30 min, as signaled by the reduction in Q_o_. Concerning the decrease in R_s_ over time, this result reveals that the CaCO_3_ salts deposited onto WE over scale formation are beneficial for the movement of charges (discharging), thus decreasing Q_s_ and boosting the EDL polarization, i.e., Q_d_, as it was indeed observed here.

In summary, the enhancement in Z over CaCO_3_ precipitation time is hypothesized to be generated by the increase in capacitive reactances (i.e., decrease in Q_o_ and Q_s_) and, principally in R_Ω_. After understanding the phenomena that drive the scale formation sensing, relative impedances (Z_rel_) were calculated from Z at different times (Z_i_) and the blank signals (Z_0_; impedance before deposition) from distinct capillaries (*n* = 3), as follows:
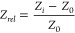
4

The values of Z were extracted at the frequency of 5 × 10^4^ Hz by offering high-sensitivity monitoring of the scale formation (see Figure 1B). The achievement of sensitive measurements at this frequency agrees with the dominance of the resistive responses over the recorded complex impedances, as this type of contribution to Z was more prominent at the highest frequencies. As exhibited in Figure 1D, Z_rel_ increased linearly with the electrodeposition time at a rate of ∼0.1 min^–1^, without reaching a stabilization plateau. This transient profile indicates not only the existence of a linear relationship between Z and the stage of scale formation but also that the SS surfaces were not totally covered by CaCO_3_ salts after 180 min of electrodeposition, as confirmed by SEM according to Figure 1E. This image also shows the three morphologies of the CaCO_3_ salts (calcite, aragonite, and vaterite), as corroborated by Raman scattering assays (Figure S5). In line with its broad applicability, one should also note that the method could gauge scale formation under different conditions by varying the flow rate (10 and 80 mL min^–1^) and temperature (25, 60, and 80 °C), and applying two off-the-shelf antiscale products (Figure S6). For the next sections, we bring the development of a two-electrode system for monitoring the spontaneous scale formation using the ideal frequency of 5 × 10^4^ Hz.

### 3.3 Two-Electrode Sensor Applied in Real Sceneries

#### 3.3.1 Continuous Analysis of Spontaneous Scale Formation

**Figure 2:**
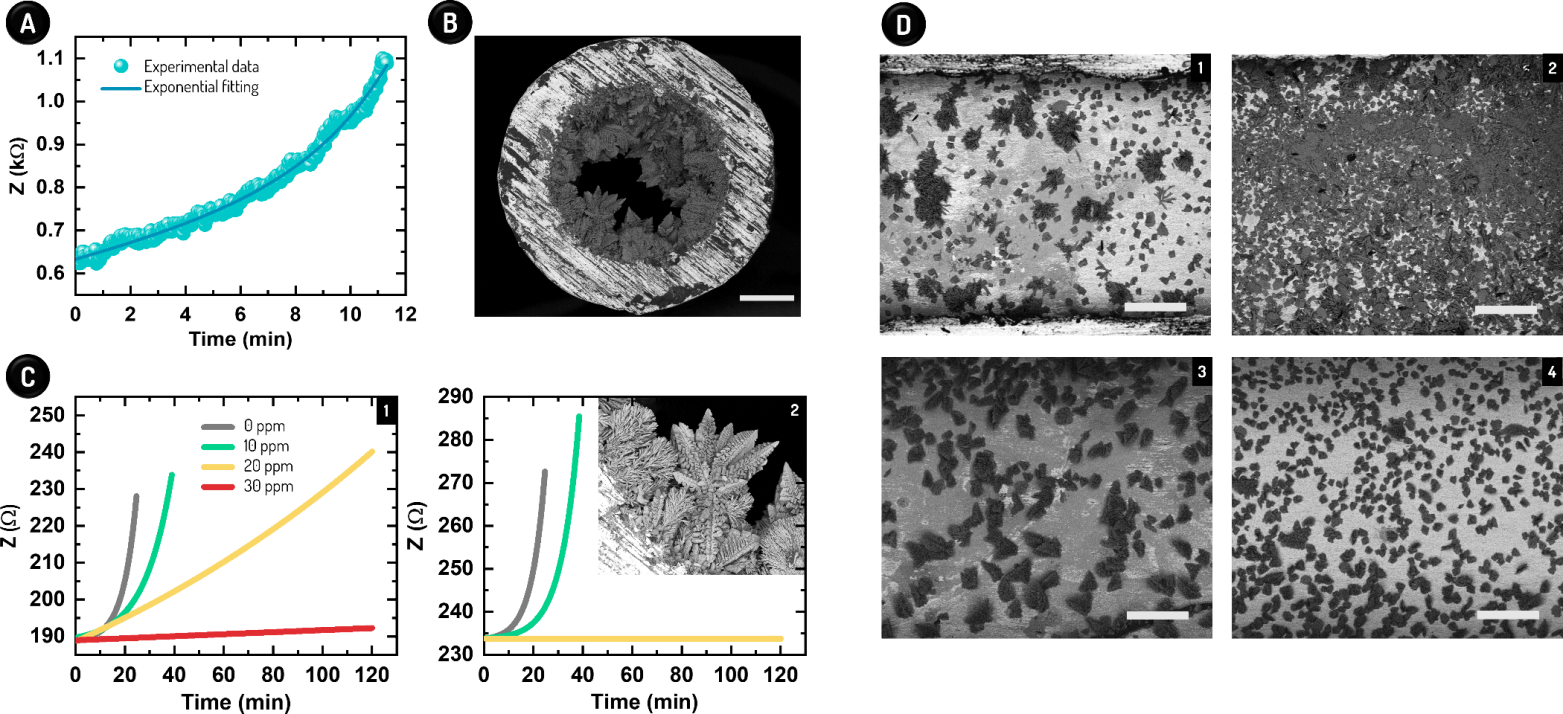
Continuous analysis of spontaneous scale formation along with the investigation of the efficacy of two scale inhibitors. (A) Z values over time and fitting based on an exponential function for kinetic evaluation. (B) SEM image of the cross-section of the capillary after 11 min of scale formation. The scale bar represents 200 μm. (C) Assessment of Products A (1) and B (2) at different concentrations. The association between concentration and curve color in (1) is also valid in (2). The inset in (2) shows a high-magnitude image of the CaCO_3_ salt on the SS capillary surface after 11 min without an antiscale product. This region was especially covered by calcite and aragonite. (D) SEM images after 900 (1,3) and 1200 s (2,4) of CaCO_3_ precipitation without (1,2) and with 20 ppm Product A (3,4). The scale bars correspond to 200 μm.

Here, we adopted a simpler version of the sensor, based on two electrodes (see Scheme 1B and Figure S2) that provided the real-time and continuous monitoring of Z over time. In this application, the sensor outlet streams were continuously discarded and the absolute Z values (at 5 × 10^4^ Hz as determined previously) represented the analytical signals of the method. This frequency provided once again the highest sensitivities. The same two prior RC equivalent circuits (see Figure 1C) were also valid in the monitoring of spontaneous scale formation. In this way, Z appeared to increase over salt precipitation time especially due to the gradual enhancement in R_Ω_ as discussed above. As can be seen in Figure 2A, such an increase in impedance was exponential. The exponential increase in Z is hypothesized to be produced by a continued scaling after SS surface coverage, with the salts protruding out in the surrounding solution and then implying an extensive ion depletion zone. The CaCO_3_ precipitation through capillary cross-section was confirmed by the SEM image of a capillary after 11 min of scale formation as exhibited in Figure 2B. This SS surface was mainly covered by calcite and aragonite.

The experimental curve (see Figure 2A) was modeled by an exponential fitting that allowed us to determine the kinetic rate constant for CaCO_3_ scale formation (*k*) on capillary as follows:
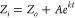
5
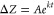
6Where ΔZ is the variation in Z, *A* is a constant related to the total Z of the system, and *t* is time. As expected, ΔZ is proportional to k, with higher values of ΔZ meaning that the scale formation is faster. We hypothesize that the *k* data depends on the nature of brines and antiscale products added into the system and on the type of SS capillary. The Eq. 6 can be employed not only to understand the salt precipitation phenomenon but also to assess the efficiency of antiscale products, as demonstrated next.

#### 3.3.2 Analysis of the Efficacy of Antiscale Products

Figure 2C presents the results for two different scale products provided by Petrobras, which were designated as Product A and Product B. The device (see Figure S3) was able to monitor the antiscale action of both products at distinct concentrations continuously, thus allowing us to determine their MICs. In agreement with TBT data obtained by Petrobras, Product B showed the best performance from our results, with MIC being estimated at 20 ppm. In this condition, Z remained constant throughout the experiment, indicating the absence of salt adsorption, as it was indeed confirmed by SEM (Figure S7). Conversely, the MIC for A is supposed to be higher than 30 ppm since this concentration was insufficient to avoid scale formation according to the Z values. Later increased as the rate of 4.6 × 10^–4^ Ω min^–1^ (R^2^: 1.00) over time. As expected, *k* decreased with the concentration of antiscale product (Figure S8), meaning that the kinetics of CaCO_3_ precipitation and then capillary blockage were sluggish after adding these chemicals. The *k* values were 1.5 and 2.3 × 10^–3^ min^–1^ for 10 ppm A and B, respectively.

SEM images attained from experiments with and without Product A confirmed the gradual CaCO_3_ precipitation-induced covering of the capillary surface (Figure S9). From Figure 2D, we can see that the SS surface blockage after 900 and 1200 s of salt precipitation is less prominent in the presence of the antiscale product. Such images combined with the kinetic Z results show that the continuously recorded Z outputs (i.e., the time-function impedance profile) are directly linked with the capillary coverage and, thus, with the scale formation stage. In this way, while the previously discussed data revealed the sensitivity of the impedimetric sensor for providing the early detection of scale formation and the batch monitoring of the effect of an antiscale product, the results presented in this section demonstrated the capability of the sensor of further affording these analyses in a real-time and continuous mode.

#### 3.3.3 Comparison of Our Sensor with Conventional TBT: Topside Scenery

We next coupled our impedimetric two-electrode sensor to two HPLC pumps, which allowed us to measure the pressure variation along experiments. In this scenario, the scale formation was simultaneously assessed through electrochemical and TBT methods by recording Z and pressure values, respectively, over time with and without antiscaling Product B at ambient pressure. With this hyphenation, a robust investigation of our sensor in comparison with a conventional approach could be made. More specifically, (i) the capacity of the sensor of signaling the moment of capillary obstruction by the scale formation and (ii) its sensitivity in probing the scale formation kinetics were scrutinized. As aforesaid, the cationic and anionic brines tested in this case triggered the precipitation of CaSO_4_, BaSO_4_, and SrSO_4_ salts onto capillary surfaces (Figure S10).

**Figure 3:**
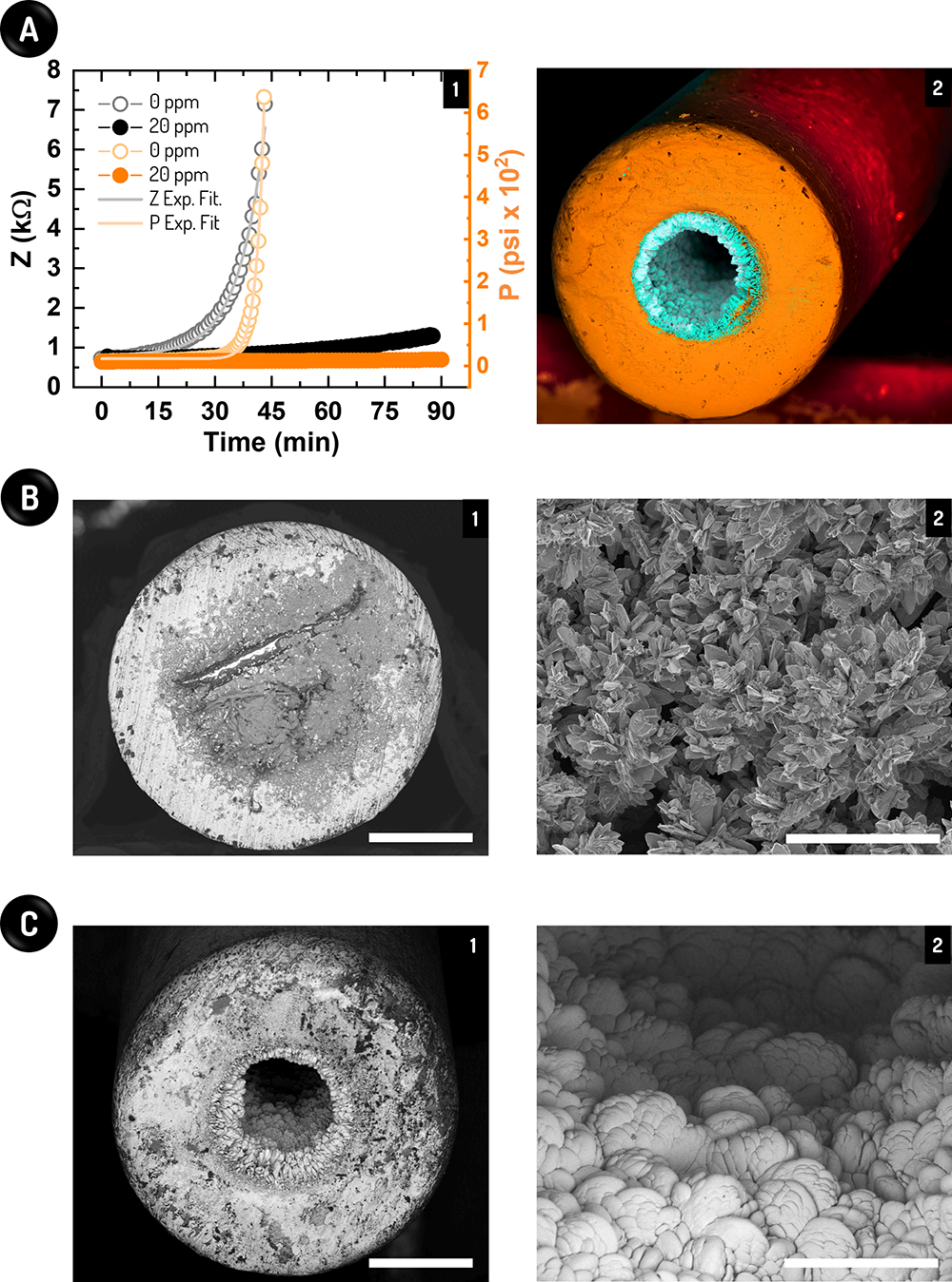
Hyphenation of our impedimetric sensor with the conventional TBT system for comparative assessment at topside conditions. (A) Responses of Z (black) and P (orange) over time in the absence (0 ppm) and presence of 20 ppm Product B (1), together with an SEM image of the capillary after 90 min with this scale inhibitor (2). In (1), the responses were fitted through an exponential function (Exp. Fit.). P in (1) means absolute pressure and the scale bar in (2) corresponds to 500 μm. (B) Cross-section (1) and amplified (2) views collected by SEM of a completely obstructed capillary after 90 min of scale formation without Product B. (C) Cross-section (1) and amplified (2) views obtained by SEM of a partially blocked capillary after 90 min using 20 ppm Product B. In (B,C), the scale bars mean 500 (1) and 100 μm (2).

Based on Figure 3A, our sensor could not only correctly appoint the time of capillary cross-section obstruction, but also continuously monitor the scale formation kinetics from its early stages. In the absence of Product B, abrupt enhancements in Z and pressure at maximum rates, i.e., 2.4 × 10^–3^ min^–1^ and 8.0 × 10^–3^ min^–1^, were both observed after 38 min of scale formation. According to SEM images of the SS capillary cross-section in Figure 3B, these changes are due to the capillary obstruction. Further, our sensor overperformed the TBT system in terms of sensitivity, as aforementioned. While the pressure data started to change only a few minutes before the obstruction of the capillary (38 min), the Z data increased continuously over time from the beginning of the experiment (see Figure 3A and Figure S11). The enhanced sensitivity of our sensor could also be witnessed along the assay with the addition of 20 ppm Product B. In this scenario, the pressure did not change over the whole experiment (90 min), meaning that this inhibitor concentration would be sufficient to prevent salt precipitation from TBT analysis. Nonetheless, as indicated by the gradual increase in the impedance values (see Figure 3A; Z increase rate of 4.4 × 10^–4^ min^–1^) and confirmed by SEM according to Figure 3C, the salt scaling still occurred under this condition but with the generation of a smoother salt structure.

In practice, the threshold for scale detection in TBT is the pressure variation of 1 psi for an experimental time 3 times the blank (i.e., the time that is needed for capillary obstruction, as evidenced by the exponential increase in pressure, in the absence of an antiscale product).^33^ However, our results revealed that the MIC inferred from these assays is lower than the effective MIC. Based on the prior TBT results, for instance, the mistaken adoption of 20 ppm as the MIC of Product B would not prevent the scale formation issue in daily applications. Remarkably, the results described in this section confirm that the impedimetric platform is more sensitive for scale formation detection than the conventional TBT system. This difference is supposed to be caused by the different principles of these methods. The variations in pressure strongly depend on the inner diameter of the capillary, whereas the Z values alter sensitively upon minimal alterations on the capillary surface and medium resistivity.^34,35^

#### 3.3.4 Comparison of Our Sensor with Conventional TBT: Subsea Scenery

**Figure 4:**
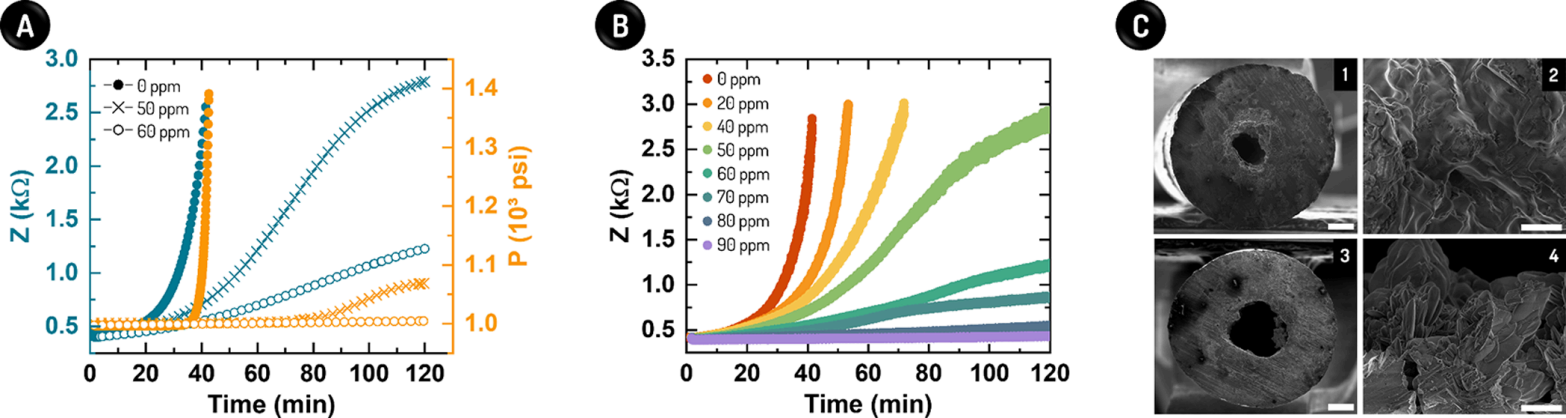
Hyphenation of our impedimetric sensor with the conventional TBT system for comparative assessment at subsea conditions. (A) Z (blue) and P (orange) values over time. The responses were once again fitted through an exponential function in both cases, i.e., without (0 ppm) and with Product C at 50 and 60 ppm, as indicated. (B) Responses for distinct concentrations of Product C, as indicated, to determine its MIC. (C) SEM images of a partially blocked capillary after 120 min using 60 (1,2) and 90 ppm (3,4) Product C. The scale bars mean 250 (1,3) and 20 μm (2,4).

We next challenged our system at a subsea scenery (1000 psi and 80 °C), where pressure plays a key role in scale formation on SS capillaries. Further, the temperature is expected to strongly impact this process because the solubility of sulfates decreases with the heating of the system. Here we apply an antiscale product termed Product C (protected formulation; it was also provided by Petrobras) in different concentrations. According to the absolute pressure and impedance results in Figure 4A, one can see that our sensor also proved to be more sensitive and accurate (for MIC prediction) than TBT at a high-pressure condition. The absolute pressure measurements led to an inaccurate MIC for Product C of 60 ppm, as revealed by SEM. From Figure 4B, the inner diameter of the SS capillary shrank by half due to the salt deposition even when adding 60 ppm Product C. In contrast, our sensor indicated the real MIC as roughly 90 ppm (see Figure 4A). In this case, nonetheless, a slight augment in Z of 5.3 Ω min^–1^ over the entire assay suggests a low but existing salt deposition. To date, this finding was once again supported by SEM imaging (see Figure 4B).

As didactically verified in the two last investigations (see Figures 3 and 4), the sensitive assessment of the scale formation processes is of pivotal relevance to avoid misleading inferences on the MIC (Table S4). Specifically, TBT can lead to negative deviations, with the predicted MIC being lower than the real value. In practice, the adoption of insufficient MICs causes the stopping of oil production due to early pipeline blockage.^36^ Conversely, it is worth underlining that the strategy based on overdosing antiscale products can increase the scale formation, as observed here (Figure S12) owing to the incompatibility between the water composition and input (i.e., Product C at concentrations higher than 90 ppm). In this regard, providing an optimum MIC is of paramount significance to prevent pipeline blockages that are critically detrimental to the entire supply chain of related inputs and products to society.

## 4 Conclusions

Using an impedimetric platform, we address the monitoring of the scale formation on SS capillaries from its early stages under real topside and subsea scenarios in the oil industry. The petrochemical industry can benefit from this platform in its long-standing and challenging task of securing ideal flow conditions, thereby sustaining the affordable supply of diverse oil-related inputs/products to society. The sensitive detection of scale formation is a mandatory attribute to ensure the accurate determination of MIC, as was indeed noted above, whereas the kinetic information provided by continuous monitoring can be used for uncovering scale formation mechanisms, thereby assisting the creation/development of antiscale chemicals.

Our method provided the continuous monitoring of the action of scale inhibitors and proved to be more sensitive than the conventional TBT system. The approach is further low-cost, basically requiring a hand-held potentiostat to be coupled to the TBT apparatus, and user-friendly. Future efforts should be focused on challenging the method at high temperatures and analyzing a large cohort of antiscale products to scrutinize the broad applicability of the approach.

## Supporting Information Available

Detailed experimental methods and characterization; two-electrode sensor in a real topside scenery–brine composition; platform design; electrical element variation; calcium quantification; surface and impedimetric studies of the CaCO3 scale; preliminary analysis of the efficacy of antiscale input; rate constant for antiscale product analysis; kinect study by SEM; EDS characterization; and comparison of our sensor with conventional TBT (PDF)
