# Supplementary material for: Sensitive Monitoring of the Minimum Inhibitor Concentration under Real Inorganic Scaling Scenarios
Source: ACS Omega. 2024 Aug 15;9(38):39724–32. doi: 10.1021/acsomega.4c04912 (PMC11425940; doi:10.1021/acsomega.4c04912)
Supplement: Supplementary file 1 — ao4c04912_si_001.pdf [file ao4c04912_si_001.pdf]

Supporting Information

Sensitive monitoring of the minimum inhibitor concentration under real inorganic scaling scenarios

Vitória M. S. Freitas[a#]; Waldemir J. Paschoalino[a#]; Luis C. S. Vieira[a]; Jussara M. Silva[b]; Bruno C. Couto[b]; Angelo L. Gobbi[a]; Renato S. Lima*[a,c,d,e]

[a]Brazilian Nanotechnology National Laboratory, Brazilian Center for Research in Energy and Materials, Campinas, São Paulo 13083-970, Brazil

[b]Leopoldo Américo Miguez de Mello Research and Development Center, Petrobras, Rio de Janeiro, RJ 21941-598, Brazil

[c]Institute of Chemistry, University of Campinas, Campinas, São Paulo 13083-970, Brazil

[d]Federal University of ABC, Santo André, São Paulo 09210-580, Brazil

[e]São Carlos Institute of Chemistry, University of São Paulo, São Carlos, São Paulo 09210-580, Brazil

*Corresponding author: renato.lima@lnnano.cnpem.br

Chemicals

NaCl, KCl, NaBr, $CaCl_2$, $BaCl_2$, $SrCl_2$, $MgCl_2$, and $NaHCO_3$ were purchased from Synth (Diadema, Brazil) and Na_2SO_4 from Merck (Darmstadt, Germany). All solutions were prepared in deionized water (Milli-Q, Millipore Corp., Bedford, MA), obtained with a resistivity of 18 MΩ cm.

Capillary tube cleaning

Stainless steel capillary tubes have impurities inside associated with the fabrication process, consequently, it is necessary to make a cleaning to ensure a uniform surface. A mixture of alkaline detergent and ketone in a 1:1 v/v ratio was used for cleaning the capillaries, followed by a rinse with deionized water, both in ultrasonic for 30 minutes each.

Heat Exchanger

The evaluation of the effects of changing brine temperatures was carried out by inserting a stainless-steel heat exchanger (HE), with a 3 cm diameter and an internal coil of 1 mm, between the pump outlet and brine inlet in the capillary. The temperature was controlled with a digital controller (Novus N1040) coupled to an internal resistance inside the HE.

Two-electrode sensor in a real topside scenery – brine composition.

Table S1. Brine composition for section 2.3.

Component	C(mg/L)
Na	27214
K	310
Mg	356
Ca	722
Ba	84
Sr	209
Cl	43732
Br	0
SO_4	1222
HCO_3	239

Platforms

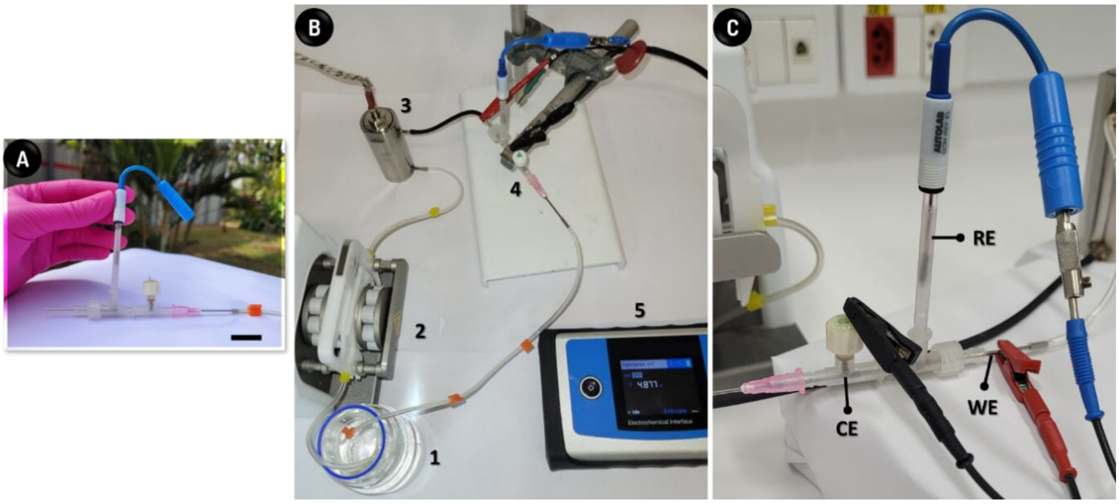

Fig. S1. Experimental apparatus for the three-electrode impedimetric sensor. **(A)** Three-electrode electrochemical sensor. **(B)** Assembly of the complete system with (1) brine, (2) peristaltic pump, (3) heat exchanger, (4) impedimetric sensor, and (5) potentiostat. **(C)** Impedimetric sensor connected in potentiostat. The scale bar in (A) corresponds to 20 mm.

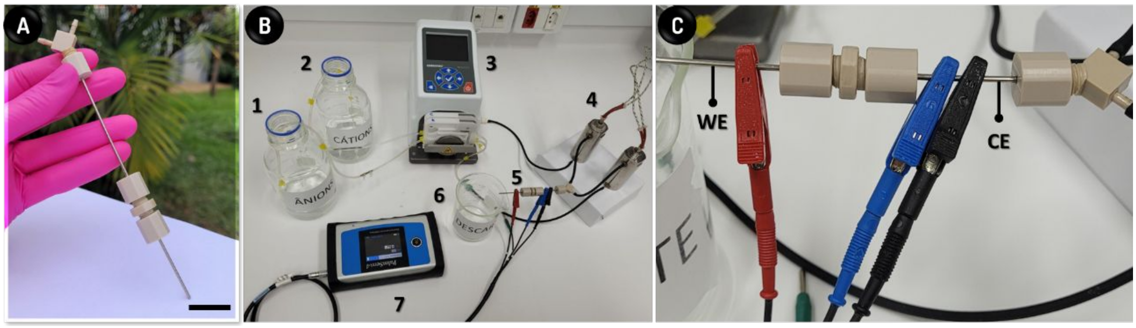

Fig. S2. Experimental apparatus for the two-electrode sensor in a real topside scenario. **(A)** Two-electrode impedimetric sensor. **(B)** Assembly of the complete system with (1) synthetic injection brine, (2) synthetic formation brine, (3) peristaltic pump, (4) heat exchangers, (5) impedimetric sensor, (6) discard, and (7) potentiostat. **(C)** Impedimetric sensor connected in potentiostat. The scale bar in (A) corresponds to 20 mm.

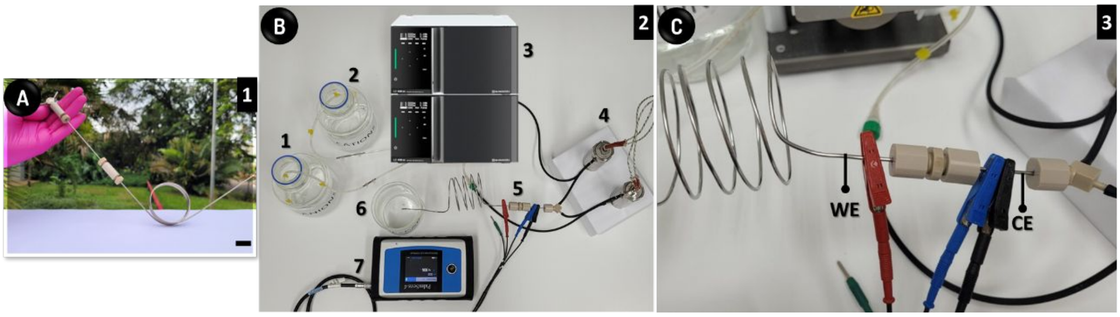

Fig. S3. Experimental apparatus for the hyphenation of our sensor with a pressure system in a real topside scenario. (A) Two-electrode impedimetric sensor. (B) Assembly of the complete system with (1) synthetic injection brine, (2) synthetic formation brine, (3) representation of HPLC pumps, (4) heat exchangers, (5) impedimetric sensor, (6) discard, and (7) potentiostat. (C) Impedimetric sensor connected in potentiostat. The scale bar in (A) corresponds to 20 mm.

Equivalent circuit parameters

Electrical elements variation

Here we correlate the variation of impedance parameters to scale formation, explaining the phenomena that happen on the SS surface. While the Z data were also governed by high values of R_o and R_s at the early stages of scaling, these parameters were reduced over time. The enhancement in Z over $CaCO_3$ precipitation time is hypothesized to be generated by the increase in capacitive reactances (i.e., decrease in Q_o and Q_s) and, principally in R_Ω. **Fig. S4** displays the values of the impedance elements over time.

Table S2. Values of n CPE parameter and error.

Time (min)	n			X^2
	Cd	Co	Ci	
**0**	0.956	0.498	-	1.20 10^{-3}
**30**	0.751	0.382	0.752	6.09 10^{-5}
**90**	0.667	0.290	0.995	4.98 10^{-5}
**120**	0.501	0.377	0.499	2.00 10^{-4}

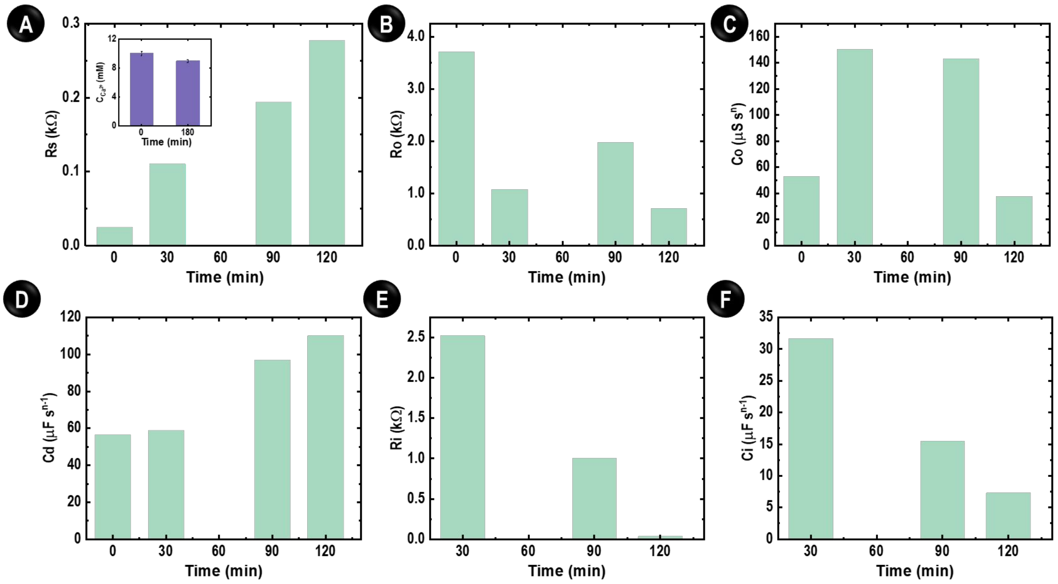

Fig. S4. Bar plots Impedance parameters over time. **(A)** Solution resistance (R_s). **(B)** Resistance of the oxide layer (R_o). **(C)** Capacitance of the oxide layer (C_o). **(D)** Double-layer Capacitance (C_d). **(E)** Resistance related to scale growth (R_i). **(F)** Capacitance of the scale layer (C_i).

Calcium quantification

A Calcium (Ca^{2+}) Ion selective electrode was used to quantify Ca^{2+} in solution before and after the experiment in a closed flow system. This experiment was performed in triplicate (n=3) with 50 mL samples under experimental conditions presented in **Table S2**.

Table S3. Experimental conditions for calcium quantification.

**Flow rate**	80 mL min^{-1}
**di**	1,0 mm
**Potential**	–1,1 V
**Temperature**	60 °C
**Electrodeposition time**	180 min

Surface and impedimetric studies of the $CaCO_3$ scale

To confirm the electrodeposition of $CaCO_3$ on the capillary surface at different flow rates and analyze the characteristics of the adsorbed salts, SEM images were obtained after electrodepositions at 0.5 and 1.0 mL min^{-1}. From **Fig. S5A**, it is possible to observe that the capillary coverage and the size of the crystals increased with the flow rate. Concerning the morphology of the salts, the three polymorphs of $CaCO_3$ salts were observed[1,2], namely, calcite (cubes), aragonite (flowers/needles), and vaterite (spheres). EDS analyses in an area with these morphologies confirmed that the $CaCO_3$ electrodeposition led to the formation of crystals only, as expected. As shown in **Fig. S5B**, their polymorphs were found to have a similar composition, mostly comprised of carbon (C), oxygen (O), and Calcium (Ca).

The $CaCO_3$ polymorphs were further investigated by Raman spectroscopy. Based on **Fig. S5C**, each morphology presented characteristic peaks at 50-400 cm^{-1} region, which are related to the lattice vibration mode of each structure[3,4,5]. 3D maps were built utilizing the peaks at 150 and 280 cm^{-1} for calcite, 150 and 210 cm^{-1} for aragonite, and 120, 280, and 300 cm^{-1} for vaterite. As exhibited in **Fig. S5D**, it is possible to distinguish the areas related to each polymorph, whose shapes agree with the literature[1,2].

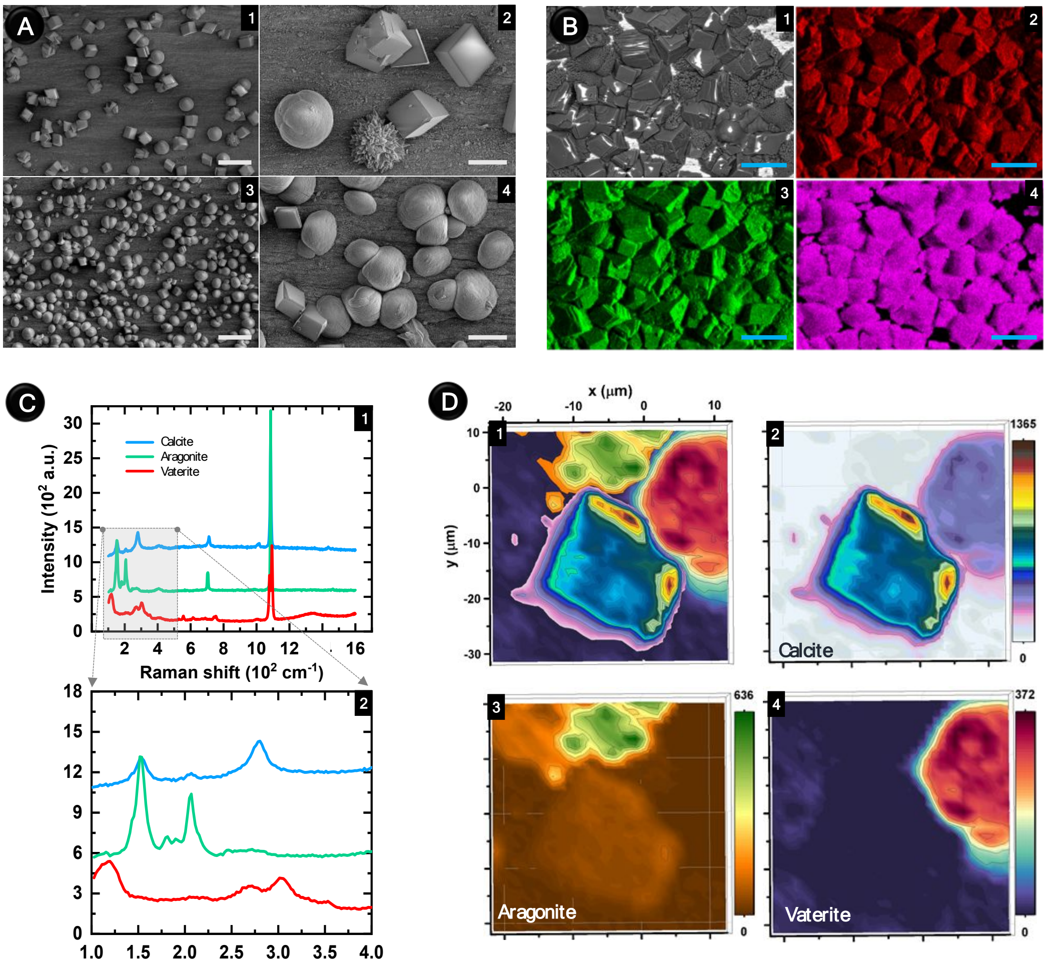

Fig. S5. Characterization of the precipitated $CaCO_3$ crystals. **(A)** SEM images at 0.5 (1,2) and 1.0 mL min^{-1} (3,4) of flow rate in a capillary with 1.0 mm id. The scale bars mean 100 (1,3) and 30 µm (2,4). **(B)** EDS graphs discriminating the elements C (red), O (green) and Ca (pink). The scale bar represents 50 µm. **(C)** Raman spectra of calcite, aragonite, and vaterite (1) and an enlarged view of these data as stressed (2). **(D)** Raman maps with recognition of the three $CaCO_3$ polymorphs.

The flow regime plays a key role in scale formation in the oil and gas pipelines[6]. In this regard, the $CaCO_3$ electrodeposition was made under the incidence of laminar flow (10.0 mL min^{-1}, with Reynolds number of ~200) and a laminar-turbulent transition flow (80.0 mL min^{-1}, with ~1700 Reynolds number). **Fig. S6A** shows SEM images of SS capillary surfaces. The scale formation at 80.0 mL min^{-1} produced a greater surface coverage and more heterogeneous $CaCO_3$ salts, presenting poorly defined shapes. This result can be unequivocally attributed to the higher number of collisions as provided by the vortices at 80.0 mL min^{-1} in comparison with the salt electrodeposition at 10.0 mL min^{-1}, boosting the nucleation spots on SS capillary walls[7].

The variation of Z_{rel} with electrodeposition time was consistent with the higher scale formation rate reached at 80.0 mL min^{-1}. More specifically, while the Z_{rel} data at 10.0 mL min^{-1} increased linearly over time from 15 min as a rate of 2.5 10^{-3} min^{-1} (R^2: 0.98), the

enhancement in signals at 80.0 mL min^{-1} occurred at a rate twice as high (5.1 10^{-3} min^{-1}, R^2: 0.96) along the whole analysis as shown in **Fig. S6B**. Moreover, the Z_{rel} value at 80.0 mL min^{-1} after 180 min of $CaCO_3$ electrodeposition was 1.4 time higher than the value at 10.0 mL min^{-1}.

The precipitation rate is also greatly affected by the temperature[8]. In particular, the solubility of $CaCO_3$ scales reduces with temperature[8], which contributes to increase the precipitation rate along the petroleum extraction pipelines. **Fig. S6C** presents the Z_{rel} values at 80.0 mL min^{-1} over the time at 25, 60 and 80 °C. As expected, the Z_{rel} variations were proportional to temperature, further signaling the direct relationship between Z and scale formation. Specifically, the rates of increase in Z_{rel} over time were calculated as (i) 9.8 10^{-3} min^{-1} (0 to 60 min) and then 3.8 10^{-3} min^{-1} (60 to 180 min) at 25 °C, (ii) 10.4 10^{-3} min^{-1} (0 to 180 min) at 60 °C, and (iii) 16.8 10^{-3} min^{-1} (0 to 180 min) at 80 °C. Linear fittings were achieved with R^2 greater than 0.98 (25), 0.95 (60), and 0.97 (80 °C). The Z_{rel} signals at the end of electrodeposition (180 min) at 80 °C were ~1.8 and ~3.5-fold higher than the values at 60 and 25 °C. The absence of stationary Z_{rel} values at 60 and 80 °C may be related to only partial scale-induced coverage of the SS capillary surfaces and/or the additional dependence of Z on the bulk nucleation as the $CaCO_3$ solubility decreases with temperature, thus further increasing R_Ω due to the ion depletion as aforesaid.

Preliminary analysis of the efficacy of anti-scale input

The prior data demonstrated that the impedimetric sensor can provide the sensitive detection of scale formation from the early stages of salt precipitation. As aforesaid, this information can be used to assess the efficiency of off-the-shelf anti-scaling products. Then, we next evaluated the performance of Product A, i.e., a commercial anti-scale input, at 10.0 and 80.0 mL min^{-1}. Experiments were conducted using the three-electrode sensor to monitor $CaCO_3$ precipitation without and with the addition of an inhibitor (20 ppm). According to the results in **Fig. S6D**, the Z sensor could monitor the action of Product A that minimized the scale formation with basis on the decrease in the Z_{rel} values. Later were revealed to be 5.6 and 1.7 times lower than the data in the absence of anti-scale chemical at the flow rates of 10 and 80.0 mL min^{-1}, respectively, after 180 min of electrodeposition. The responses after adding Product A practically achieved stationary profiles from 60 min at both flow rates, indicating an extensive coverage of the SS surfaces by the $CaCO_3$ salts.

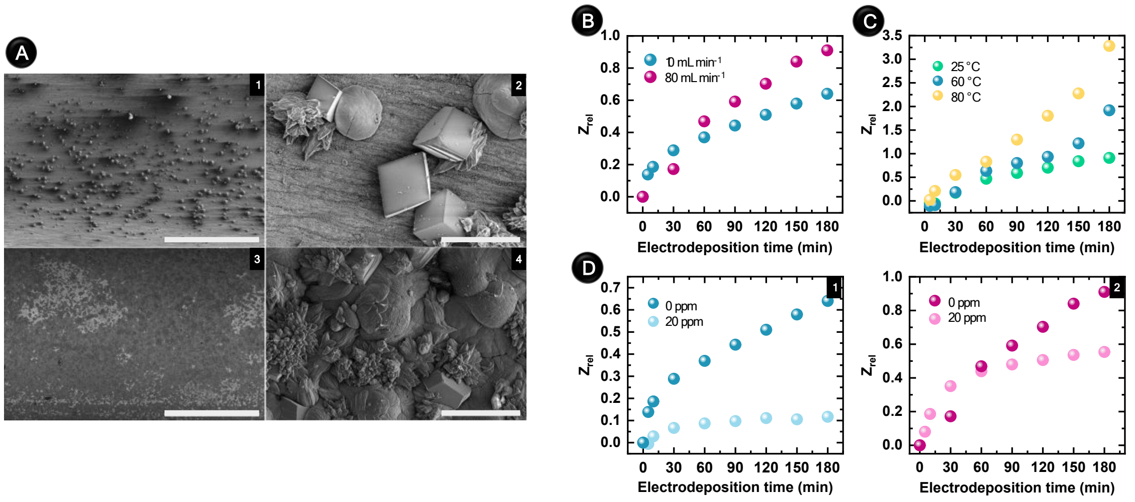

Fig. S6. Surface and impedimetric studies of the $CaCO_3$ scale and preliminary analysis of the efficacy of an anti-scale input. **(A)** SEM images for flow rates of 10.0 (1,2) and 80.0 mL min^{-1} (3,4). The scale bars mean 500 (1,3) and 30 µm (2,4). **(B)** Z_{rel} *vs* time results for experiments at 10.0 and 80.0 mL min^{-1}. **(C)** Plots of Z_{rel} *vs* time for experiments at 25, 60, and 80 ºC. **(D)** Z_{rel} *vs* time in the absence and presence of 20 ppm Product A at 10.0 (1) and 80.0 mL min^{-1} (2).

Tests using 20 ppm of anti-scale Product B

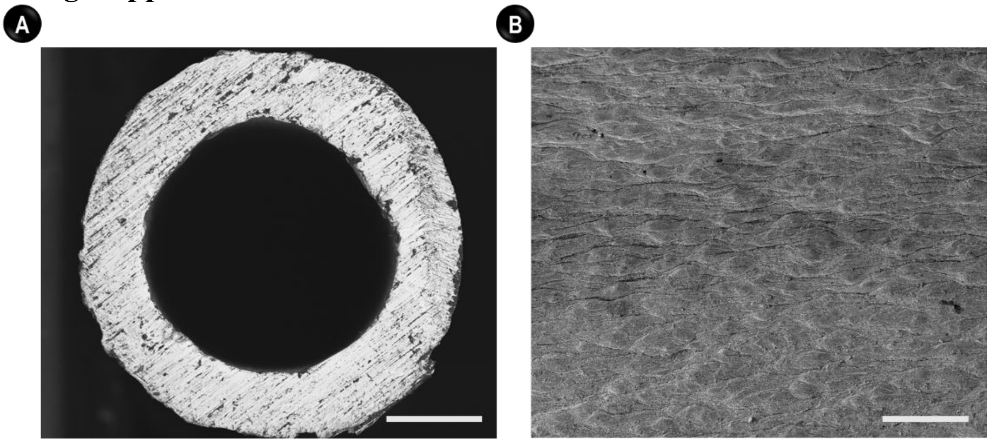

Fig. S7. SEM images of tests using 20 ppm of anti-scale product B in the two-electrode system. **(A)** Transversal section of the capillary and **(B)** inner wall of the capillary. Scale bar represents 250 and 50 mm, respectively.

Rate constant for anti-scale product analysis

We demonstrate that ΔZ is proportional to k, with higher values of Z meaning that the scale formation is faster. We also hypothesize that the values of k depend on the nature of brines, the presence of anti-scale products, and the type of SS capillary, as shown in **Fig. S8**.

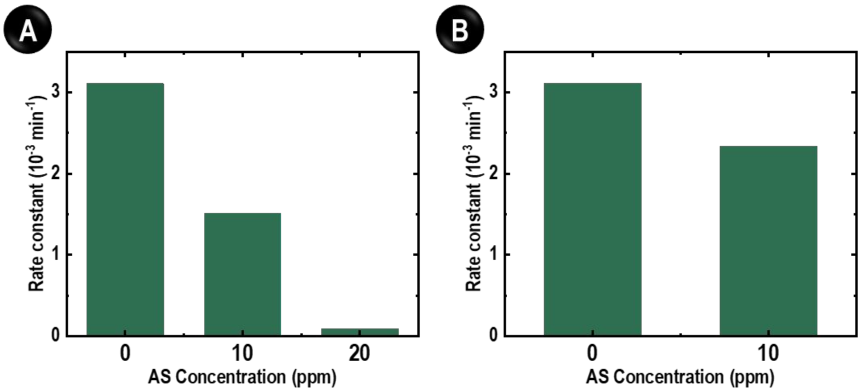

Fig. S8. Rate constant of the scale formation with and without the presence of **(A)** Product A and **(B)** Product B.

Kinect study by SEM

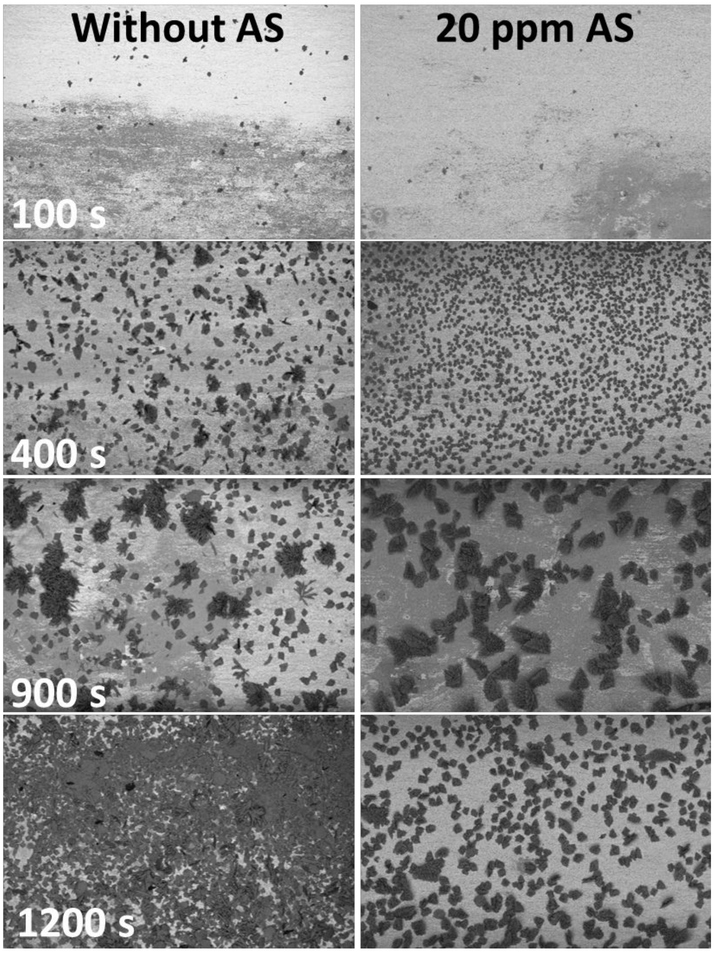

Fig. S9. SEM images of CaCO3 deposition over time with and without the presence of 20 ppm of Product A.

EDS characterization

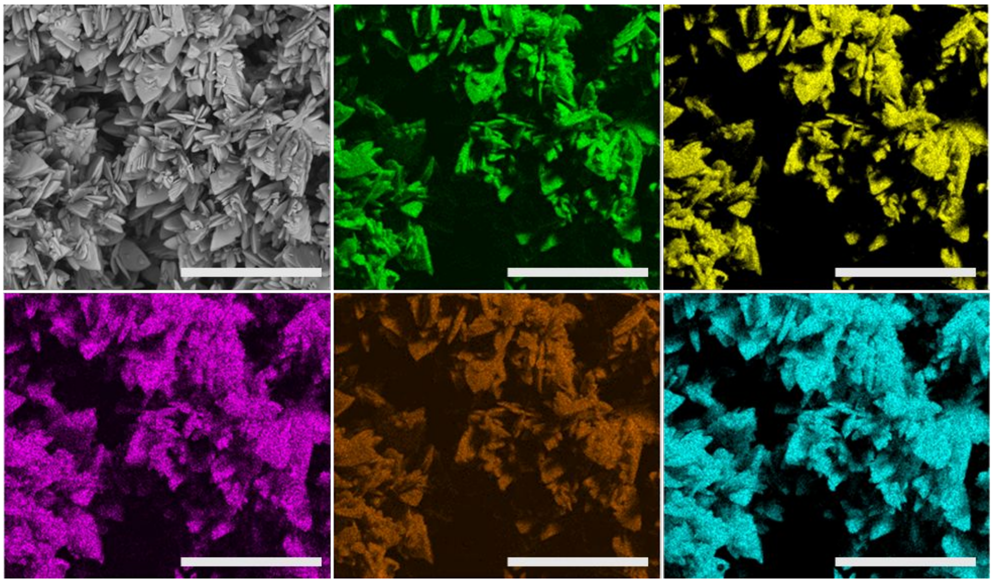

Fig. S10. EDS measurements separating the elements O (green), S (yellow), Ca (pink), Sr (orange) and Ba (cyan). The scale bar represents 60 mm.

Comparison of our sensor with conventional TBT

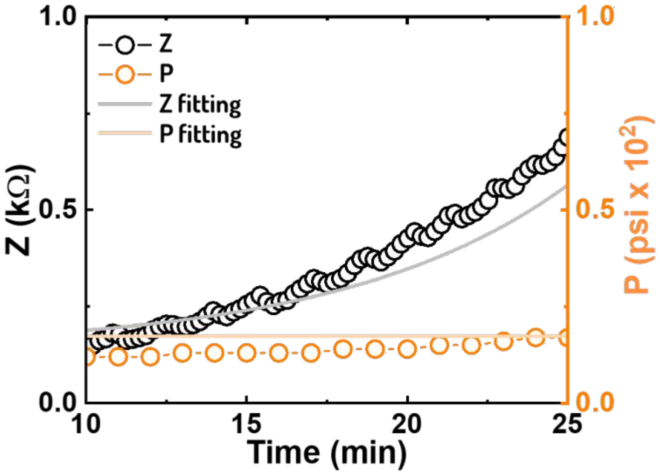

Fig. S11. Z and P values over time response fitted with an exponential function without Product B.

Table S4. Summary of TBT and Impedance MIC for a subsea and topside scenery.

Scenery	Method	MIC (mg/L)
Topside	Pressure	20
	Impedance	30
Subsea	Pressure	60
	Impedance	90

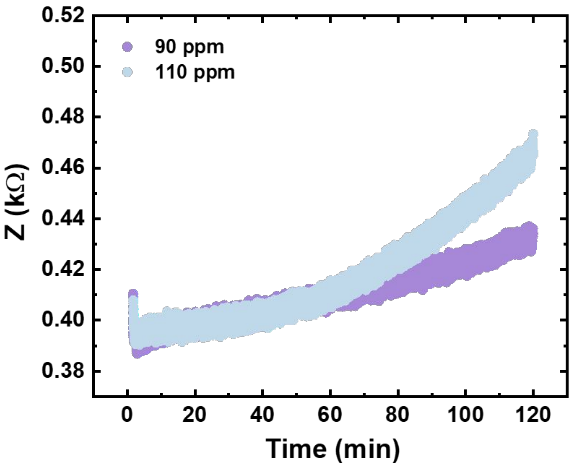

Fig. S12. Z over time for subsea scenery in the presence of 90 and 110 ppm of Product C.

REFERENCES

(1) Ševčík, R.; Šašek, P.; Viani, A. Physical and Nanomechanical Properties of the Synthetic Anhydrous Crystalline CaCO3 Polymorphs: Vaterite, Aragonite and Calcite. *J Mater Sci* **2018**, *53* (6), 4022–4033. https://doi.org/10.1007/s10853-017-1884-x.

(2) Blue, C. R.; Giuffre, A.; Mergelsberg, S.; Han, N.; De Yoreo, J. J.; Dove, P. M. Chemical and Physical Controls on the Transformation of Amorphous Calcium Carbonate into Crystalline CaCO3 Polymorphs. *Geochim Cosmochim Acta* **2017**, *196*, 179–196. https://doi.org/10.1016/j.gca.2016.09.004.

(3) Karoui, H.; Riffault, B.; Jeannin, M.; Kahoul, A.; Gil, O.; Ben Amor, M.; Tlili, M. M. Electrochemical Scaling of Stainless Steel in Artificial Seawater: Role of Experimental Conditions on CaCO3 and Mg(OH)2 Formation. *Desalination* **2013**, *311*, 234–240. https://doi.org/10.1016/j.desal.2012.07.011.

(4) De La Pierre, M.; Carteret, C.; Maschio, L.; André, E.; Orlando, R.; Dovesi, R. The Raman Spectrum of $CaCO_3$ Polymorphs Calcite and Aragonite: A Combined Experimental and Computational Study. *J Chem Phys* **2014**, *140* (16), 164509. https://doi.org/10.1063/1.4871900.

(5) Dandeu, A.; Humbert, B.; Carteret, C.; Muhr, H.; Plasari, E.; Bossoutrot, J. M. Raman Spectroscopy – A Powerful Tool for the Quantitative Determination of the Composition of Polymorph Mixtures: Application to CaCO3 Polymorph Mixtures. *Chem Eng Technol* **2006**, *29* (2), 221–225. https://doi.org/10.1002/ceat.200500354.

(6) Olajire, A. A. A Review of Oilfield Scale Management Technology for Oil and Gas Production. *J Pet Sci Eng* **2015**, *135*, 723–737. https://doi.org/10.1016/j.petrol.2015.09.011.

(7) Moxey, D.; Barkley, D. Distinct Large-Scale Turbulent-Laminar States in Transitional Pipe Flow. *Proceedings of the National Academy of Sciences* **2010**, *107* (18), 8091–8096. https://doi.org/10.1073/pnas.0909560107.

(8) MacAdam, J.; Parsons, S. A. Calcium Carbonate Scale Formation and Control. *Rev Environ Sci Biotechnol* **2004**, *3* (2), 159–169. https://doi.org/10.1007/s11157-004-3849-1.
